# A remarkable new species of *Picolistrus* Majer, 1990 (Coleoptera, Dasytidae) from Yunnan, China

**DOI:** 10.3897/BDJ.13.e156603

**Published:** 2025-05-30

**Authors:** Jialin Miao, Haoyu Liu, Junbo Tong, Xingke Yang, Yuxia Yang

**Affiliations:** 1 Key Laboratory of Zoological Systematics and Application, College of Life Sciences, Hebei University, Baoding, China Key Laboratory of Zoological Systematics and Application, College of Life Sciences, Hebei University Baoding China; 2 Hebei Basic Science Center for Biotic Interaction, Hebei University, Baoding, China Hebei Basic Science Center for Biotic Interaction, Hebei University Baoding China; 3 Key Laboratory of Zoological Systematics and Evolution, Institute of Zoology, Chinese Academy of Sciences, Beijing, China Key Laboratory of Zoological Systematics and Evolution, Institute of Zoology, Chinese Academy of Sciences Beijing China

**Keywords:** alpha taxonomy, dasytid beetles, morphology, identification key

## Abstract

**Background:**

*Picolistrus* Majer, 1990, is a small genus belonging to the subfamily Listrinae of the family Dasytidae (Coleoptera, Cleroidea). Currently, it comprises five known species: *P.inhirsutus* (Pic, 1922) and *P.gemmatus* Plonski, 2016 from China; *P.palleatus* Majer, 1990, *P.brunneus* Majer, 1990 and *P.helferi* Majer, 1990 from Myanmar.

**New information:**

A new dasytid species of the genus *Picolistrus* Majer, 1990 has been discovered from Yunnan, China and is here described under the name of *P.flavoapicalis* sp. nov. The description is illustrated with habitus, ultimate abdominal tergite and sternite and genitalia of both sexes. The generic diagnosis is updated. A key for identification and a distribution map of all *Picolistrus* species are provided.

## Introduction

The genus *Picolistrus* Majer, 1990 is currently classified within the subfamily Listrinae Majer, 1990 of the beetle family Dasytidae (Majer 1990, Mayor 2007). There are 10 genera included in the Listrinae, which are characterised by the following characters: pronotum without sublateral lines, all tibiae present with short spinules around apices, tarsomeres 3 and 4 subequal in size, tarsal claws symmetrical, with a pair of completely membranous appendages about as long as claws, appendages more or less attached to claws; abdominal sternite VIII bilobed, median process absent or present and floating; base of tegmen not bent ventrally, median lobe tube-like (Majer 1990, Gimmel and Mayor 2024). Adults of *Picolistrus* can be easily distinguished from all other Asiatic genera by the short and cylindrical body, upper body surface densely covered with very fine, short and recumbent pubescence and lateral margins of pronotum and elytra fringed with erect hairs and all tibiae with fixed spines along the length of the outer face; differential diagnosis given by Majer (1990).

In the original description by Majer (1990), *Picolistrus* was designated with *Dasytiscusinhirsutus* Pic, 1922 as its type species, along with three additional species. After that, only one more species was added (Plonski 2016). At present, this genus comprises five species distributed in China and Myanmar (Majer 1990, Plonski 2016). In this study, we report on an interesting species discovered in Yunnan, China. Its distinctive external morphology allows for easy differentiation from all other members and it is described as new to science below. Our findings will enhance the understanding of the species diversity amongst dasytid beetles in China.

## Materials and methods

In this study, we adhere to the conventional taxonomic classification of dasytid beetles as a separate family, Dasytidae (Majer 1994, Majer 1996, Mayor 2007, Bocakova et al. 2012, Constantin 2015, Plonski 2016, Miao et al. 2024, Miao et al. 2025), rather than regarding them as a subfamily within Melyridae (Gimmel et al. 2019, Gimmel and Mayor 2024). This study primarily focuses on the species descriptions of *Picolistrus*, despite the ongoing debates regarding its higher classification, which falls outside the scope of this research. The studied specimens are deposited in the Institute of Zoology, Chinese Academy of Sciences, Beijing, China (IZAS).

The specimens were initially soaked in water for softening, followed by the separation of their abdomens. The separated abdomens were then immersed in a 10% sodium hydroxide (NaOH) solution and heated at a constant temperature for several minutes using a metal bath. Once the fat had dissolved, they were transferred to a Nikon SMZ1500 stereomicroscope for the dissection of the pygidium, abdominal sternite VIII and genitalia. To facilitate observation, the spiculum gastrale, tegmen and median lobe were isolated respectively. The ovipositor was stained with haematoxylin. Subsequently, the dissected genitalia were placed on a glass slide with glycerol and photographed using a Leica M205A stereomicroscope before being stored in glycerol for preservation. A Canon EOS 80D digital camera was used to capture images of habitus which were later processed using Helicon Focus 7 software. Adobe Photoshop CC 2019 version 20.0.4 was utilised for editing in plate preparation. The body length was measured from the anterior margin of the head to the elytral apices and the width at the humeri. The terminology of genital segments follows Majer (1994) and that of genitalia follows Gimmel and Mayor (2019). In addition, some other terms of male genitalia are proposed in the present study as follows: apical limb: distal part of median lobe, generally slender; basal limb: basal part of median lobe, generally stout; subapical ring: an orifice of tegmen, which is a space delimited by membranes of the subparts of tegmen.

The distribution map was prepared by ArcMap 10.8 and edited in Photoshop CC 2019 20.0.4, based on distribution information from relevant literature (Majer 1990, Plonski 2016) and the studied material.

## Taxon treatments

### Picolistrus

Majer, 1990

E733D3C0-D6F6-5642-BDF9-F65DC1233356

Picolistrus Majer, 1990 - Majer 1990: 377.
Dasytiscus inhirsutus Pic, 1922

#### Diagnosis

**Figure 1.**
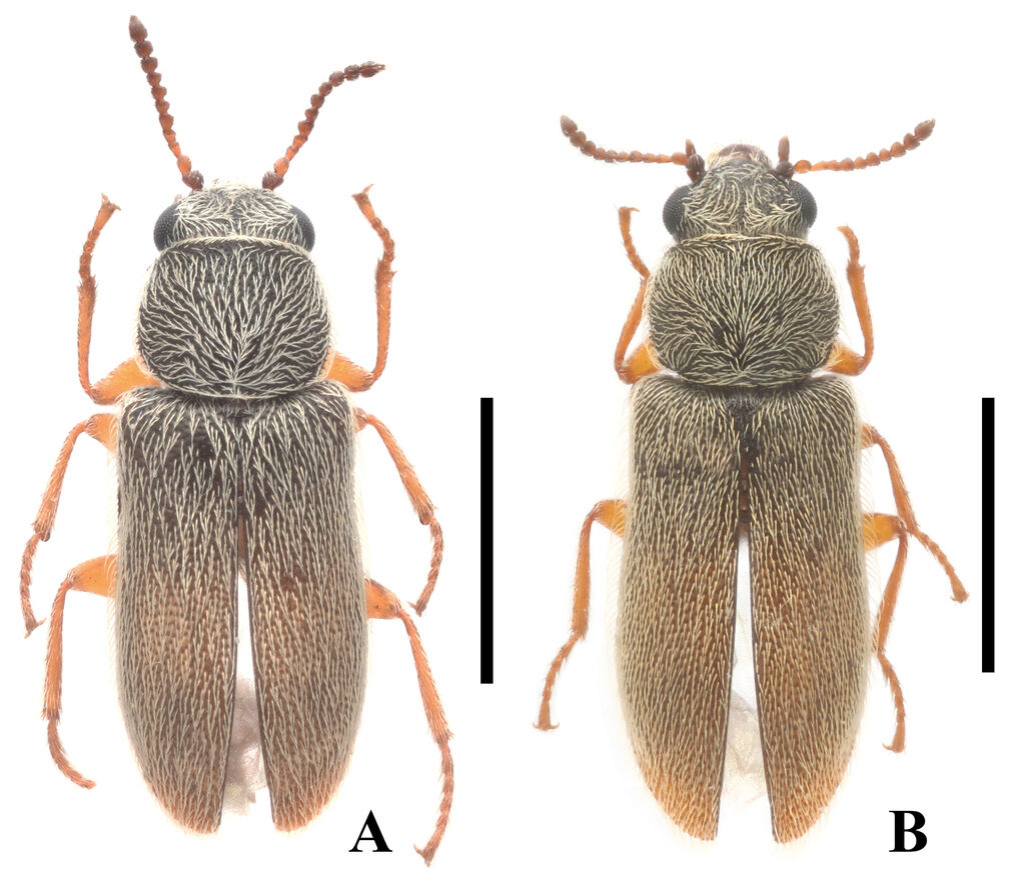
Habitus of *Picolistrusflavoapicalis* Y. Yang & Miao, sp. nov., dorsal view: **A** male; **B** female. Scale bars: 1.0 mm.

**Figure 2.**
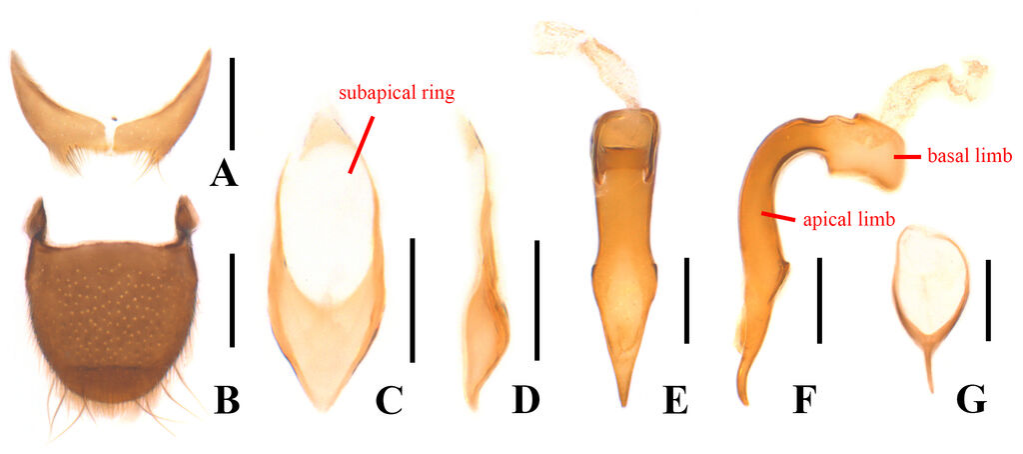
*Picolistrusflavoapicalis* Y. Yang & Miao, sp. nov., holotype, male: **A** abdominal sternite VIII, ventral view; **B** pygidium, dorsal view; **C** tegmen, ventral view; **D** tegmen, lateral view; **E** median lobe, ventral view; **F** median lobe, lateral view; **G** spiculum gastrale, ventral view. Scale bars: 0.2 mm.

**Figure 3.**
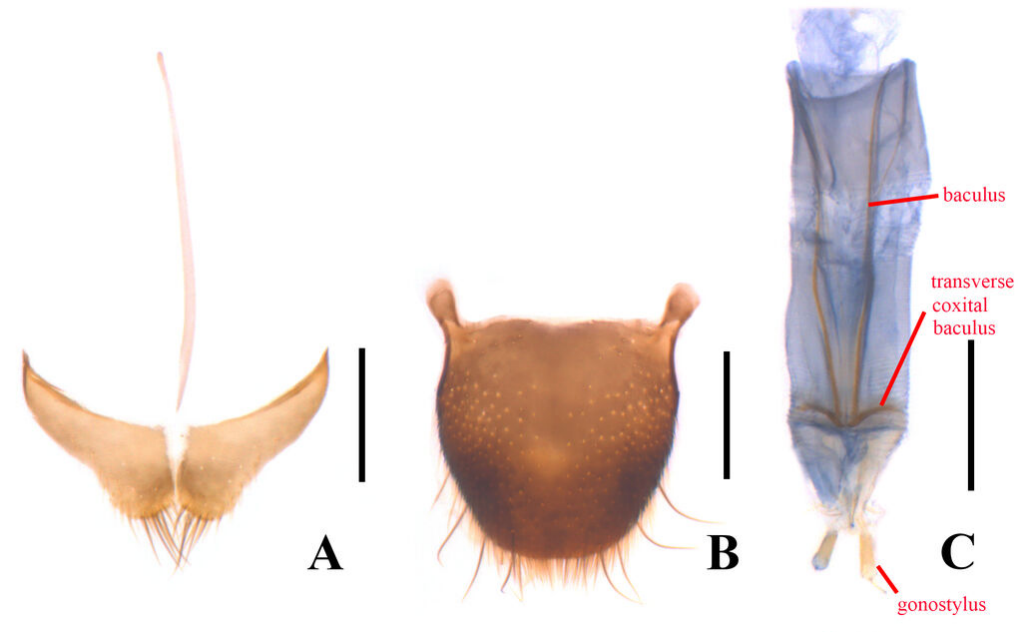
*Picolistrusflavoapicalis* Y. Yang & Miao, sp. nov., paratype, female: **A** abdominal sternite VIII, ventral view; **B** pygidium, dorsal view; **C** ovipositor, ventral view. Scale bars: 0.2 mm.

Body small-sized and stout, 1.3–3.0 mm in length. Upper body surface densely covered with very fine, short and recumbent pubescence. Lateral margins of pronotum and elytra fringed with erect hairs obliquely directed backwards (Fig. 1). Antennomeres 6–9 at most as wide as long, mostly transverse. All tibiae with fixed spines along the length of the outer face in both sexes and males with enlarged fixed spurs at the apical inner base of one or all tibiae (pro-, meso- and/or metatibiae); tarsi shorter than tibiae, tarsomeres 1–4 wider than long; tarsal claws symmetrical, with a pair of completely membranous appendages, which are about as long as claws. Abdominal sternite VIII completely separated, with median process often expressively reduced or absent in males (Fig. 2A). Apex of tegmen without any setae, not or slightly protruding apically (Fig. 2C); apical limb of median lobe elongate triangular at distal half and sharp at apex in ventral view (Fig. 2E), basal limb strongly bent ventrally, at an angle of at least 90˚ with the apical limb, apical limb prominently angled near middle, tapered apically and apex slightly incurved ventrally in lateral view (Fig. 2F). Spiculum gastrale (Fig. 2G) Y-shaped. Ovipositor (Fig. 3C) stout and membranous, gonostylus long and nearly cylindrical, transverse coxital baculus short and bisinuate, baculus long and oblique.

#### Distribution

**Figure 4.**
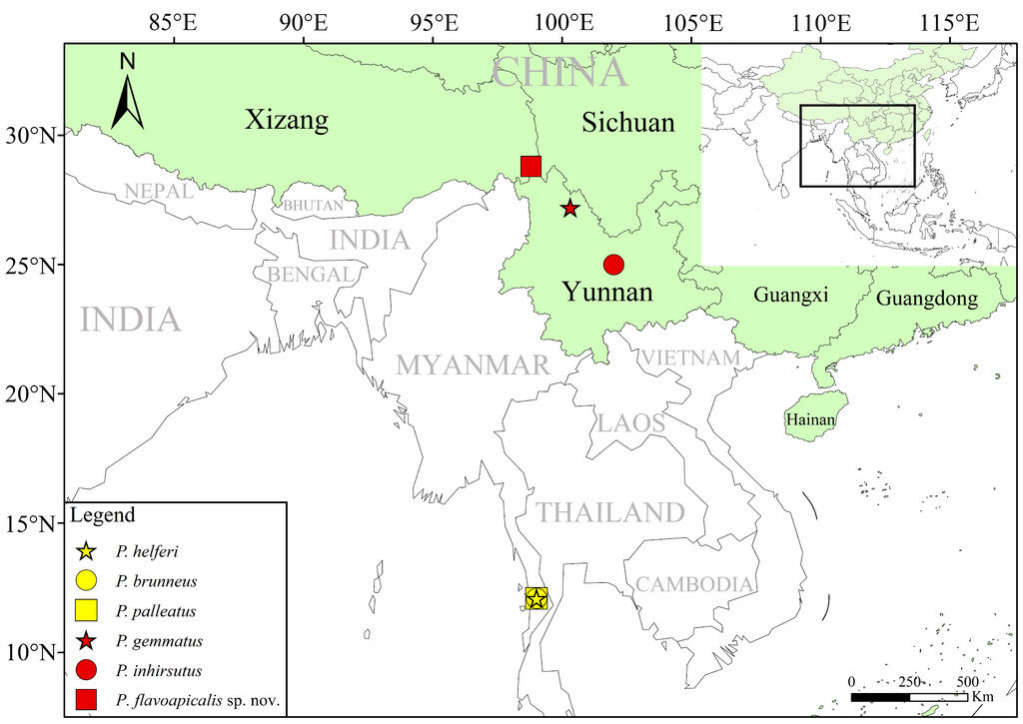
Distribution map of all *Picolistrus* species.

China (Yunnan), Myanmar (Tenasserim) (Fig. 4).

### Picolistrus flavoapicalis

Y. Yang & Miao sp. nov.

E4C06286-62AC-5BAB-9BDD-48336AFB92FD

#### Materials

- **Type status:** Holotype. **Occurrence:** recordedBy: Shuyong Wang; sex: 1 male; lifeStage: adult; occurrenceID: B4EAE4E6-FD29-5102-B4D4-792FE13B6E74; **Location:** country: China; stateProvince: Yunnan; county: Dêqên; locality: Meilishi village; verbatimElevation: 2200 m; **Event:** year: 1982; month: 7; day: 19; **Record Level:** institutionID: Institute of Zoology, Chinese Academy of Sciences; institutionCode: IZAS
- **Type status:** Paratype. **Occurrence:** recordedBy: Shuyong Wang; sex: 5 females; lifeStage: adult; occurrenceID: EF201593-6473-52D1-8101-CC060C451120; **Location:** country: China; stateProvince: Yunnan; county: Dêqên; locality: Meilishi village; verbatimElevation: 2200 m; **Event:** year: 1982; month: 7; day: 19; **Record Level:** institutionID: Institute of Zoology, Chinese Academy of Sciences; institutionCode: IZAS

#### Description

**Male** (Fig. 1A). Body length 2.25 mm, width 0.78 mm.

Body black with slight lustre, elytral apices brownish-yellow. Antennomeres 1 blackish-brown, 2–11 brownish and gradually darkened towards apex. Legs brownish-yellow, with femora, tibiae and tarsomeres more or less darkened at apices. Body surface densely and shallowly punctate, densely covered with short and recumbent light yellow pubescence, pubescence on pronotum directing medially and posteriorly towards the mid-point of posterior margin.

Eyes moderately prominent, head width across eyes feebly wider than anterior margin of pronotum. Antennae extending to posterior 1/8 length of pronotum when inclined, with antennomeres 1 nearly conical, 2 ellipsoidal, 3–5 triangular and longer than wide, 6–10 globular, 11 fusiform, 1.5 times as long as wide.

Pronotum transverse and 1.2 times as wide as long, widest behind middle, anterior and posterior margins slightly arcuate, lateral margins arcuate with sparse crenation, anterior and posterior angles widely rounded. Elytra subparallel-sided, 1.9 times longer than humeral width, 2.3 times longer than pronotum, elytral edges fine, but distinct, rounded at apices. Protibia and mesotibia each with a concave, lamellate fixed spur at apical inner base.

Abdominal sternite VIII (Fig. 2A) strongly transverse and bilobed, rectangularly emarginate at posterior margin, acute at antero-lateral angles, present with a very short median process, surface covered with a few erect setae near posterior margin. Pygidium (Fig. 2B) slightly longitudinal, 1.2 times longer than wide, feebly narrowed posteriorly, slightly arcuate at anterior margin, not emarginate at posterior margin, with antero-lateral angles protruding and acute at apices, surface covered with a few long erect setae and short erect hairs along posterior margin.

Aedeagus: tegmen (Fig. 2C) with an elliptic subapical ring extending beyond middle from apical 1/8, both apex and base approximately triangular in ventral view; median lobe with apical limb more than 9.0 times longer than basal width in lateral view (Fig. 2F). Spiculum gastrale (Fig. 2G) with basal trunk shorter than apical branch.

**Female** (Fig. 1B). Similar to male, but body larger, 2.49–2.54 mm in length, 0.82–0.83 mm in width. Antennae slightly shorter, extending to posterior 1/4 length of pronotum when inclined, with antennomeres 6–10 slightly transverse. Pronotum 1.3 times as wide as long. Elytra distinctly dilated across apical third, 2.1 times longer than humeral width, 2.5 times longer than pronotum. Each tibia with two ordinary, sharp spurs at apical inner base. Abdominal sternite VIII (Fig. 3A) bilobed, with antero-lateral angles acute, median process very slender and distinctly extending beyond antero-lateral angles. Pygidium (Fig. 3B) shallowly emarginate in middle of anterior margin, not emarginate at posterior margin, with antero-lateral angles obviously protruding and round at apices.

#### Diagnosis

This species can be easily distinguished from all other species of *Picolistrus* by the bicoloured elytra, which are black and brownish-yellow at apices (Fig. 1), tegmen sharp at apex in ventral view (Fig. 2C) and apical limb of median lobe slender, more than 9.0 times longer than basal width (Fig. 2F). In comparison, the elytra of all others are uniformly black or brown, except for *P.palleatus* Majer 1990, whose elytra are testaceous with a large shadowy spot around black scutellum. Tegmen of all others are broadly rounded (Majer (1990): fig. 71; Plonski (2016): fig. 3) or slightly emarginate at apex (Majer (1990): fig. 52). Apical limb of median lobe is less elongate, at most 6.0 times longer than basal width in others (Majer (1990): figs. 53, 69, 72 and 73; Plonski (2016): fig. 2).

#### Etymology

The species epithet is derived from the Latin *flavus* (yellow) and *apicalis* (of or belonging to an apex), referring to its elytra with brownish-yellow apices.

#### Distribution

China (Yunnan) (Fig. 4).

## Identification Keys

### Key to the species of the genus *Picolistrus* Majer, 1990

**Table:** 

1	Elytra bicoloured	2
–	Elytra unicolour	3
2	Elytra mostly black and brownish-yellow at apices; tegmen approximately triangular at apex in ventral view (Fig. 2C); apical limb of median lobe more than 9.0 times longer than basal width (Fig. 2F)	*P.flavoapicalis* Y. Yang & Miao, sp. nov.
–	Elytra testaceous with large shadowy spot around black scutellum; tegmen broadly rounded at apex in ventral view (Majer (1990): fig. 71); apical limb of median lobe no more than 5.0 times longer than basal width (Majer (1990): fig. 72)	*P.palleatus* Majer, 1990
3	Body brown; antennomere 10 as long as wide; elytral edges extremely fine and nearly invisible	*P.brunneus* Majer, 1990
–	Body black; antennomere 10 distinctly wider than long; elytral edges fine, but distinct	4
4	Legs black, rufescent at base of tibiae and tarsi, sometimes uniformly rufopiceous; tegmen with a subapical ring extending from apical 1/7, slightly emarginated at apex in ventral view (Majer (1990): fig. 52)	*P.inhirsutus* (Pic, 1922)
–	Legs uniformly testaceous; tegmen with a subapical ring extending from apex, broadly rounded at apex in ventral view (e.g. Plonski (2016): fig. 3)	5
5	Upper surface of body with bluish metallic lustre; abdominal sternite VIII present with a very short median process in male; apical limb of median lobe straight dorsally at distal half in lateral view (Majer (1990): fig. 73)	*P.helferi* Majer, 1990
–	Upper surface of body without metallic lustre; abdominal sternite VIII absent with any median process in male (Plonski (2016): fig. 5); apical limb of median lobe arcuate dorsally at distal half in lateral view (Plonski (2016): fig. 2)	*P.gemmatus* Plonski, 2016

## Supplementary Material

XML Treatment for Picolistrus

XML Treatment for Picolistrus flavoapicalis
